# DIVINE–pilot trial: a phase 2 multicentre, randomised pilot trial of pharmacotherapy and physical activity monitoring conducted in women with recent gestational diabetes and increased risk of type 2 diabetes recruited from tertiary referral hospitals in Australia

**DOI:** 10.1136/bmjopen-2025-107551

**Published:** 2025-12-12

**Authors:** Angela Xun-Nan Chen, Vivian Y Lee, Katherine Donges, Chris Giancas, Blake Angell, Belinda Parmenter, Helen L Barrett, Amanda Henry, Anushka Patel, Clare Arnott

**Affiliations:** 1 Cardiovascular Programme, The George Institute for Global Health, Sydney, New South Wales, Australia; 2 Department of Diabetes and Endocrinology, Westmead Hospital, Westmead, New South Wales, Australia; 3 Cardiovascular Programme, The George Institute for Global Health, Barangaroo, New South Wales, Australia; 4 Sydney, Sydney, New South Wales, Australia; 5 The George Institute for Global Health, Barangaroo, New South Wales, Australia; 6 UNSW Medicine and Health Lifestyle Clinic, University of New South Wales, Sydney, New South Wales, Australia; 7 Discipline of Sport and Exercise Science, School of Health, University of the Sunshine Coast - Sunshine Coast Campus, Sippy Downs, Queensland, Australia; 8 Obstetric Medicine, Royal Hospital for Women, Randwick, New South Wales, Australia; 9 Discipline of Women’s Health, School of Clinical Medicine, University of New South Wales Medicine and Health, Sydney, New South Wales, Australia; 10 Women’s Health Programme, The George Institute for Global Health, Barangaroo, New South Wales, Australia; 11 Department of Obstetrics and Gynaecology, St George Hospital, Kogarah, New South Wales, Australia; 12 Department of Cardiology, Royal Prince Alfred Hospital, Camperdown, New South Wales, Australia; 13 Department of Cardiology, St Vincent’s Hospital Sydney, Darlinghurst, New South Wales, Australia

**Keywords:** Diabetes in pregnancy, Overweight, Obesity, Diabetes Mellitus, Type 2, Randomized Controlled Trial, Cardiovascular Disease

## Abstract

**Introduction:**

Women who develop gestational diabetes mellitus (GDM) have a 60% lifetime risk of developing type 2 diabetes mellitus (T2D), which is already elevated within the first decade following childbirth. Despite the impact of lifestyle interventions to reduce long-term T2D risk in women with previous GDM, successful implementation of lifestyle interventions remains a barrier. Metformin is recommended for adults at increased risk of developing T2D; however, there is limited evidence of tolerability in the early postpartum period. Glucagon-like peptide 1 receptor agonists (GLP-1 RA) are effective at improving glycaemic status and body weight. However, GLP-1 RA have not been evaluated in the postpartum population. Finally, physical activity monitors may support behaviour changes related to physical activity to reduce long-term risk of T2D but are yet to be studied following GDM.

**Methods and analysis:**

This will be a multicentre, randomised, open-label interventional pilot study. Using a 2×2 factorial design, we will examine the feasibility and acceptability of a pharmacotherapy intervention and a physical activity intervention in women with previous GDM at increased risk of developing T2D. Participants will be recruited from tertiary referral hospitals in Australia and will be randomised to receive either metformin alone or in combination with a GLP-1 RA and subsequently randomised to either a physical activity intervention involving activity monitor use, or usual care for 6 months, followed by a 6-month follow-up period. Primary feasibility outcomes include the acceptability and safety of the metformin and GLP-1 RA as measured through pill and injection counts, acceptability questionnaire and adverse events.

**Ethics and dissemination:**

This trial is registered with the Australian and New Zealand Clinical Trials Registry (Registration Number: ACTRN12624001253594). This trial has received ethics approval from the South Eastern Sydney Local Health District Human Research Ethics Committee (Approval Number: 2024/ETH00042, protocol version v1.1, 28/02/2025).

**Trial registration number:**

Australian and New Zealand Clinical Trials Registry, Registration Number: ACTRN12624001253594.

**STRENGTHS AND LIMITATIONS OF THIS STUDY**

- Robust evaluation using prospective, randomised, open, blinded evaluation approach.
- Inclusion of a population at high risk for type 2 diabetes mellitus (T2D) that has been neglected in pharmaco-prevention trials to date.
- The 6-month follow-up period after the intervention allows for assessment of sustained effects on long-term cardiovascular risk factors, body composition and behavioural changes. This will be crucial for evaluating the longer-term effectiveness and acceptability of the proposed intervention.
- Lack of participant blinding, which may introduce performance or detection bias.
- This pilot trial is not powered to reliably detect effects on the development of T2D. However, the findings will provide important data on feasibility, acceptability and preliminary data to inform the design of future adequately-powered trial of T2D prevention.

## Introduction

 Once thought to be fully reversed after childbirth, gestational diabetes mellitus (GDM) is now firmly established as an independent risk factor for type 2 diabetes mellitus (T2D) and cardiometabolic disease in women.^1^ GDM prevalence continues to increase; in Australia, 1 in five pregnancies is currently affected by GDM.^2^ Up to 40% of women with GDM have persisting dysglycaemia in the first year postpartum,^3^ conveying a 10–20-fold increase in risk for developing T2D within 5 years.^1^ There is a clear need for early and effective interventions to reduce the risk of T2D in women with a history of GDM.^4^

Recent meta-analyses evaluating the impact of lifestyle interventions for prevention of T2D in women with a history of GDM reported a 19%–24% risk reduction in incidence of T2D compared with standard care.^5 6^ However, when studies were restricted to interventions delivered within 5 years postpartum, the evidence was equivocal.^7^ The largest randomised trial to date (LIVING study, n=1601) did not show improvements in glycaemic status following a 12-month lifestyle intervention in GDM-affected women who were on average 7 months postpartum.^3^ Challenges in the implementation of lifestyle modifications in the early postpartum period^8^ highlight the need for novel adjuncts to current models of care.

Studies evaluating pharmacological interventions to prevent T2D in women with previous GDM have shown some benefits with a range of drug classes including metformin, thiazolidinediones, incretins and dipeptidyl peptidase-4 inhibitors.^9^ Metformin remains the class with the strongest evidence for T2D prevention.^10^ In the Diabetes Prevention Programme, metformin alone reduced the incidence of T2D by 50.4% compared with placebo in women with a history of GDM.^11^ However, less than 5% of the general at-risk prediabetic population are being prescribed metformin for T2D prevention,^12^ and there is a lack of data regarding metformin prescription rates in women with previous GDM and pre-diabetes.

Weight gain is the single strongest predictor of long-term T2D risk in women with a history of GDM.^13^ Glucagon-like peptide 1 receptor agonists (GLP-1 RA) are a class of drug shown to reduce HbA1c with additional weight loss benefits in patients with and without T2D.^14^,^16^ While emerging data shows GLP-1 RA use in overweight women with previous GDM is associated with weight reduction, long-term medication tolerability is a concern.^17^ Further, there are concerns for accelerated muscle mass loss associated with GLP-1 RA use^18 19^ and rapid weight regain with GLP-1 RA cessation,^20^ warranting the need for further studies with use for any population.

Wearable activity monitors increase physical activity in healthy adults,^21^ sedentary older adults^22^ and in clinical populations such as people living with T2D, overweight and obesity.^23^ These devices track activity levels, such as step count and intensity minutes (ie, time spent in multiple intensities according to heart rate). Activity monitors may increase physical activity among high-risk postpartum women with previous GDM, with emerging evidence supporting their feasibility.^24^ This is particularly timely given growing recognition of the need for complementary interventions to overcome implementation barriers related to sustained behavioural change.

## Rationale

Given the uncertainty regarding the efficacy, safety and acceptability of both pharmacotherapy and activity monitor use in the postpartum setting, there is a need for further high-quality data. This pilot trial will generate data regarding the feasibility, acceptability and tolerability of such interventions in women with recent GDM and heightened risk of progressing to T2D. In addition, this study will generate preliminary data to facilitate the design of an adequately powered efficacy trial for the prevention of T2D.

## Methods and analysis

### Study design

**Figure 1:**
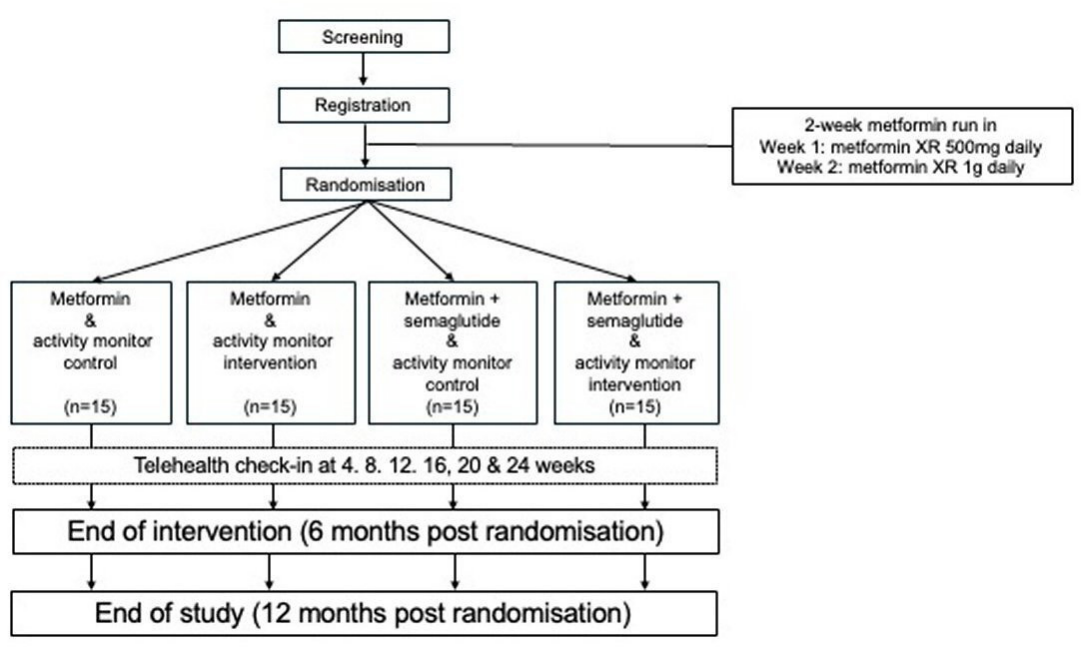
DIVINE-pilot study schema.

This protocol was developed according to the Standard Protocol Items: Recommendations for Interventional Trials 2013 checklist (online supplemental appendix 1).^25^ This prospective, multi-centre, open label, randomised, interventional trial will evaluate the feasibility, safety and acceptability of metformin versus a combination of metformin and semaglutide (GLP-1 RA) in women within 5 years of childbirth of a GDM-affected pregnancy, who are at increased risk of developing T2D. Using a 2×2 factorial design (figure 1), participants will also be randomised to a physical activity intervention or usual care control group.

### Study participants

#### Inclusion criteria

Women aged 18 years and older, 3–60 months postpartum who experienced GDM in their most recent pregnancy, with persisting postpartum dysglycaemia and body mass index (BMI) ≥25 kg/m^2^ will be eligible to participate. Evidence of postpartum dysglycaemia will be defined as having fasting plasma glucose 6.1–6.9 mmol/L and/or 2-hour blood glucose 7.8–11.0 mmol/L on 75 g oral glucose tolerance test (OGTT) performed within the past 12 months; and/or glycated haemoglobin (HbA1c) ranging between 6.0%–6.4%, performed within the past 12 months and at least 3 months postpartum. Other inclusion criteria include eGFR ≥45 mL/min/1.73 m^2^, willingness to take a pregnancy test prior to starting intervention and during follow-up, willingness to wear an activity monitor (Garmin) for the duration of the study, access to smart mobile phone or tablet to enable Garmin Connect App use and the ability to provide written informed consent. Women who are already using metformin for dysglycaemia (but not T2D) are eligible for study participation.

#### Exclusion criteria

Women will not be eligible to participate in the trial if they are currently pregnant, breast-feeding or actively planning pregnancy; have known T2D, type 1 diabetes mellitus or any other forms of current diabetes; have a history of bariatric surgery (eg, gastric bypass, gastric sleeve, gastric banding) or if there is known intolerance to metformin or a current absolute contraindication to metformin therapy. Additional exclusion criteria include known intolerance or allergy to semaglutide, current use of a semaglutide or another GLP-1 RA, or known personal or family history of medullary thyroid cancer or multiple endocrine neoplasia type 2 gene. Further, potential participants will be excluded from the trial if they have received treatment with any other medication for the indication of overweight or obesity within the last 90 days or have previous or planned (during the trial period) obesity treatment with surgery or weight loss device. Individuals who are unwilling to use International Council for Harmonisation of Technical Requirements for Pharmaceuticals for Human Use defined ‘highly effective’ contraception, unable to participate in physical activity or have known inability to give informed consent to participate due to severe active mental health issues or major developmental disability as identified through the maternity database will also be excluded from trial participation.

### Recruitment and screening activities

#### Recruitment

Participants will be recruited by two tertiary teaching hospitals in metropolitan Australia. Three strategies will run concurrently to identify potential participants to ensure timely recruitment. First, potentially eligible women enrolled in a recent cohort study (DIVINE-NSW),^26^ who have provided written consent to be contacted for follow-up studies, will be approached. Second, potentially eligible women who gave birth at the trial sites will be identified using medical records. Thirdly, the study team will disseminate information to clinical networks outside the recruitment area to seek referrals of potential participants to a study site.

#### Informed consent and screening

Potentially eligible participants will be contacted by the study team and provided with detailed information about the study. They will have the opportunity to discuss any questions and will be given sufficient time to consider their participation before providing consent. Written informed consent will be obtained in accordance with International Council for Harmonisation Guideline for Good Clinical Practice from all potential participants, prior to conducting any trial procedures, including screening assessments. This consent will be reaffirmed at the registration visit to confirm consent to trial participation and randomisation.

### Study procedures

#### Registration and trial enrolment

At the registration visit, a member of the study team will obtain the participant’s demographic and past medical history and confirm blood investigations that demonstrate evidence of persisting dysglycaemia. Baseline data including anthropometrics (weight, height and waist circumference), blood pressure and heart rate will be collected (table 1). Participants will complete Quality of Life (EQ-5D-5L, online supplemental appendix 2) and Self-reported physical activity (IPAQ-short, online supplemental appendix 3) questionnaires.

**Table 1:** Summary of study timeline and assessments

	Registration −2 weeks	Run-in −2 to 0 weeks	Randomisation 0 weeks	Follow-up 4, 8, 12, 16, 20, 24 weeks (telehealth check-ins)	End of intervention 26 weeks	End of trial 52 weeks
Medical history	X					
Demographics	X					
Anthropometrics	X				X	X
Body composition (DEXA)			X		X	X
BP and HR	X				X	X
Quality of life (EQ-5D-5L)	X				X	X
Self-reported physical activity (IPAQ-short)	X				X	X
Brief medication questionnaire				X	X	
Exercise physiologist review			X			
Pathology test						
75 g oral glucose tolerance test	X				X	X
Fasting glucose	X				X	X
HbA1c	X				X	X
Urine pregnancy test	X		X*	X*		
Medications						
Drug tolerability and adherence		X	X	X	X	
Review of concomitant medications	X	X	X	X	X	X
Activity monitor						
Review of daily activity				X	X	
Acceptability of activity monitor questionnaire					X	X
Safety						
Serious adverse events		X	X	X	X	X
Safety outcomes		X	X	X	X	X

* In participants randomised to metformin and semaglutide.

BP, blood pressure; DEXA, dual-energy X-ray absorptiometry; EOI, end of intervention; HbA1c, glycosylated haemoglobin; HR, heart rate.

#### Active run-in

During a 2-week non-blinded active run-in, participants will commence metformin SR 500 mg daily for 7 days, followed by metformin SR 1000 mg daily for 7 days. If metformin SR 1000 mg daily is not tolerated, down-titration to 500 mg daily will be allowed. If potential participants are already taking metformin, up-titration to 1000 mg daily will be undertaken. If the metformin dose is higher than 1000 mg daily, there will be no change to the dose. Participants already taking immediate-release metformin will be switched to a slow release formulation.

#### Randomisation and blinding

Participants who have adhered (≥80% adherence) and tolerated metformin run-in will be randomised to a pharmacological intervention study arm and subsequently randomised into physical activity intervention study arm.

Randomisation will be performed by a central study statistician using web-based software, concealed from participants and study staff, and will be stratified by study site. The central study team and outcome assessors will be blinded to allocated groups. Unblinding will not be necessary as unblinded personnel at the corresponding sites will be responsible for clinical decisions.

Baseline body composition scan using dual-energy X-ray absorptiometry (DEXA) will be performed, and a urinary pregnancy test will be completed to ensure a negative result prior to dispensing study drugs (table 1). A visit with an accredited exercise physiologist (AEP) will occur within 2 weeks of randomisation.

### Study interventions

The trial design is summarised in figure 1. After the metformin run-in, participants will be randomly assigned in a factorial design to the two intervention comparisons, that is the pharmacological intervention of metformin alone versus metformin and semaglutide and the physical activity intervention of activity monitor intervention versus activity monitor control. Thus, the intervention arms will be: (a) Metformin+activity monitor intervention, (b) Metformin+activity monitor control, (c) Metformin and semaglutide+activity monitor intervention and (d) Metformin and semaglutide+activity monitor control.

#### Pharmacological intervention

##### Metformin+semaglutide arms

Metformin SR 1000 mg daily (or individualised tolerable dose) will be taken throughout the trial intervention period of 6 months. Semaglutide will be up-titrated every 4 weeks, starting at 0.25 mg weekly to a maximum weekly tolerated dose of 1 mg weekly.

##### Metformin arms

Metformin SR 1000 mg daily (or individualised tolerable dose) will be taken throughout the trial intervention period of 6 months.

### Physical activity intervention

All the participants will be given an activity monitor (Garmin Vivosmart 5) to wear throughout the intervention period. They will also attend one individual session with an AEP and will be provided with nutritional information from Diabetes Australia in written form from site study staff.^27^

#### Activity monitor intervention arms

Participants will have full access to the activity monitor and the Garmin Connect App which provides detailed information on their activity levels. Participants will receive individualised AEP advice to increase frequency and intensity of physical activity and reduce sedentary behaviour to reach the National Health and Medical Research Council guidelines recommendations on physical activity in adults (150 min of moderate-intensity exercise or equivalent).^28^ The key aims for the physical activity group will be to:

1. Increase physical activity by 10% each week until they reach the National Health and Medical Research Council guidelines on physical activity in adults (150 min of moderate-intensity exercise or equivalent).^28^ If a participant’s physical activity level already meets current National Health and Medical Research Council guidelines, they will be encouraged to maintain these target levels and, where possible, increase further if feasible.
2. Focus more on higher intensity exercise, if possible, by focusing on activity minutes on the Garmin device (Only if found to be safe to do so through screening with the).
3. Move (for at least 10 min) when the move bar is triggered (triggered if inactive for an hour)—walking continuously for a few minutes, the bar will reset and disappear from the screen. A full inactivity metre can take up to 200 continuous steps to reset completely.
4. Personalised recommendations based on their preferences—set up step and activity minute goals—activity minutes are automatically collected by their HR measures on the Garmin even if they are not recorded as an exercise session.
5. Focused on the little changes in everyday life (ie, feeling stronger while doing everyday things, eg, playing with kids) and changes in feeling (eg, feeling energised for the day, doing something for self, getting a good night’s sleep).

#### Activity monitor control arms

Participants will receive a device showing time only to maintain participant blinding to activity levels. The physical activity control group will have Garmin Connect App set up on their mobile phone for data collection purposes but will be instructed not to open the App. Participants will be provided with general advice by the AEP.

### Follow-up period

All participants will be followed up for a further 6 months following termination of study intervention. All data related to study outcomes will be collected (table 1). Those on metformin prior to the intervention will be advised to continue such therapy as prescribed by their General Practioner (GP). All participants will be asked to avoid use of any other weight loss pharmacotherapy (including semaglutide) during the follow-up. All participants will be allowed to keep their activity monitor, with full access to the App, and asked to continue using them at their discretion.

### Study follow-up and assessments

#### Telehealth follow-up

Telehealth (telephone or video call) follow-up visits will be conducted at weeks 4, 8, 12, 16, 20 and 24. This will enable assessment of pharmacological and physical activity intervention tolerability and compliance using the Brief Medication Questionnaire (online supplemental appendix 4) and Acceptability of activity monitor questionnaire (Appendix 5) respectively and provide opportunities to up-titrate semaglutide dose for applicable participants. The occurrence of any study outcomes and adverse events (AEs) in all participants, as well as adherence to contraception and urinary pregnancy test results in those randomised to metformin+semaglutide will be reviewed (table 1).

#### End of intervention visit

All data related to study outcomes will be collected (table 1). This will include measurements of weight, waist circumference, body composition (DEXA), blood pressure and resting heart rate. Blood investigations including fasting glucose, 75 g OGTT and HbA1c will be performed. Evaluation of quality of life (EQ-5D-5L, online supplemental appendix 2) and acceptability of activity monitor questionnaire (online supplemental appendix 5) will be administered. A pill count will be performed and data collected on medication adherence, AEs and attitudes to medication use. Physical activity data will be collected through Garmin Connect App.

#### End of intervention visit

All data related to study outcomes will be collected (table 1).

### Outcomes

#### Primary feasibility outcomes

1. Acceptability and tolerability of metformin as assessed by pill count at the end of intervention period (key feasibility outcome).
2. Acceptability and tolerability of semaglutide as assessed by injection count during the intervention period (key feasibility outcome).
3. Acceptability of activity monitor assessed by the acceptability questionnaire following the intervention and follow-up period (key feasibility outcome).

#### Secondary outcomes

1. The change in absolute body weight (kg) from baseline to 6 months.
2. Mean daily dose of metformin achieved during the intervention period.
3. Mean weekly dose of semaglutide achieved during the intervention period.
4. Permanent discontinuation of therapy.
5. Change in fasting plasma glucose (mmol/L) from baseline.
6. Change in HbA1c (%) from baseline.
7. Change in proportions with dysglycaemia (impaired glucose tolerance, impaired fasting glucose or T2D), as assessed by 2-hour OGTT blood glucose (mmol/L) from baseline.
8. Change in systolic and diastolic blood pressure (mm Hg) from baseline.
9. Change in BMI (kg/m^2^) from baseline.
10. Change in waist circumference (cm) from baseline.
11. Change in body composition (body fat mass and muscle mass, %) from baseline.
12. Participant quality of life (EQ-5D-5L survey).
13. Change in mean activity levels (minute/week) from baseline.
14. Change in self-reported physical activity level from baseline.
15. Change in mean daily step count from baseline.

#### Safety outcomes

Safety outcome relates to AEs. The timing, duration, severity, management and consequences of AEs associated with the trial drugs and physical activity monitor use will be documented. Their relationship to the use of the trial drugs and physical activity monitor will be identified.

### Statistical analysis

#### Statistical methods

Appropriate descriptive statistics will be provided for all study variables including demographic and baseline characteristics. Mean, median, SD, minimum and maximum will be used to summarise continuous variables. Counts and percentages will be used to summarise categorical variables. Continuous secondary endpoints will be analysed using linear regression or similar. Secondary outcomes examining differences in proportions will be analysed using logistic regression or similar.

Efficacy and safety analyses will be based on the intention-to-treat principle, with participants analysed according to the study group to which they were randomised. The primary analyses are not anticipated to include imputation for missing data. A secondary analysis of safety outcomes will be performed using treatment received.

#### Sample size

This pilot trial will be used to identify an appropriate upper limit SD estimate to avoid under-powering the main study.^29^ The Type I error rate (α) of the main study will be 0.05. The anticipated SD estimate from the pilot trial is 18.6 kg,^30^ and the confidence level for the upper confidence limit of the SD will be 95%. To detect a mean difference in body weight of 8.7 kg in the main study with 90% power, the optimum pilot trial sample size is 55. Anticipating an 8% dropout rate, 60 participants will be enrolled to obtain a final sample size of 55 subjects. The pilot trial sample size was computed using PASS 2025, V.25.0.1. Dropout is defined as exit from the trial for whatever reason prior to the completion of the study (ie, end of intervention visit).

### Health economic evaluation

A modelled economic evaluation will be conducted using a health system perspective to assess the relative cost-effectiveness of metformin monotherapy and metformin+semaglutide compared with the current recommended measures of diet and lifestyle alone. We will adapt the UKPDS outcomes model, a computer simulation model designed to estimate the long-term impact of health interventions for people with T2D,^31^ with data collected through the trial to produce locally relevant estimates of cost-effectiveness by extrapolating trial findings of the impact of the intervention on weight and HbA1c to improvements in life expectancy and quality of life.

Health service utilisation data will be collected through the trial and estimates of downstream healthcare costs will be generated based on published estimates of Australian costs for treatment of diabetes and diabetes-related complications. Metformin and semaglutide will be costed using private prescription prices as neither medication is PBS-approved for the current study indication. Cost-effectiveness results will be expressed as the incremental cost per quality adjusted life year in the intervention group versus comparator.

### Data monitoring committee

A data safety monitoring committee has been formed and consists of three independent researchers with expertise in clinical trials, gestational diabetes and pharmacotherapy interventions. They will meet at regular intervals throughout the trial with a focus on safety outcomes and AEs. Given this is a pilot trial, no interim analyses are planned.

## Ethics and dissemination

This trial was registered with the Australian and New Zealand Clinical Trials Registry (Registration Number: ACTRN12624001253594). This trial has received ethics approval from the South Eastern Sydney Local Health District Human Research Ethics Committee (Approval Number: 2024/ETH00042, protocol version v1.1) and has been ratified by the University of New South Wales on the 16 June 2025.

Trial outcomes will be presented at national and international scientific conferences and published in international peer-reviewed journals in the broader area of Maternal Health, Diabetes and Cardiovascular Disease Prevention. Authorship for publications will be based on the International Committee of Medical Journal Editors criteria.

## Discussion

The increasing prevalence of GDM and its associated risk for the development of long-term health complications warrants urgent clinical need to find effective treatments to reduce the long-term T2D risk in these women.^5^ Although intensive lifestyle intervention is recognised as a foundational pillar to mitigate long-term T2D risk, we and others have shown that implementation of behavioural change remains a barrier, especially during the early postpartum period, to reducing long-term cardiometabolic risk in women with previous GDM.^4 7^

This multi-centre, open label, randomised, interventional trial will provide important insight into the potential impact of pharmacological treatments and physical activity. Monitors for the reduction of long-term T2D risk in women with previous GDM. Outcomes of this trial may be a precursor for an important paradigm shift in our overall approach to T2D risk reduction in women with previous GDM. The key strength of the proposed trial is in taking a multi-modality approach that addresses lifestyle and behaviour change via the physical activity tracker and combines this with proven pharmacological interventions that are well known to reduce T2D risk in the general population.^15 16^ Although evidence suggests that the effects of GLP-1RAs tend to diminish after discontinuation,^30^ their use during periods of elevated risk may offer a valuable window of opportunity. Combining the pharmacotherapy with the physical activity approach will provide structured support to address the implementation barriers related to sustainable lifestyle modifications, which could serve as an important strategy for long-term risk reduction.

This trial aims to identify acceptable and potentially effective preventive interventions that can be safely delivered earlier in the woman’s life course. Prior studies of pharmacological interventions have been largely conducted 10–15 years following the diagnosis of GDM.^10^ Further, behaviour change in relation to T2D and weight gain prevention is complex and generally more effective when individuals have realistic expectations and are able to set small and achievable goals.^32^

To our knowledge, this is the first trial to evaluate the impact of metformin and/or semaglutide in this high-risk population of postpartum women with previous GDM. It further provides evidence on the feasibility of wearable physical activity monitors in enhancing behaviour change to improve long-term risk profiles in this population. Key strengths of trial design include its multi-centre, randomised design and the inclusion of a further 6-month follow-up period following the end of the trial intervention. This allows us to understand the short-term impact of ceasing pharmacological interventions in the patient population. As there is emerging evidence that lean body mass loss may be accelerated with the use of GLP-1 RA,^23^ a further trial strength is the incorporation of DEXA body composition scans in the pilot trial to better understand the impact of GLP-1 RA use in women of childbearing age, a study population that is often left out of GLP-1 RA trials. We have also included a health economic analysis as part of the trial analysis. This will be crucial to better understand if the proposed interventions are cost-effective, as this will have major implications on the broader generalisability and uptake of such interventions.

The proposed pilot trial is not without its limitations. Mainly, the lack of participant blinding for GLP-1 RA. However, to ensure reliable and accurate outcome measures, individuals assessing the outcomes will be blinded to the group allocations. Placebo was considered but not sought for the pilot trial due to the additional constraints related to supply of placebo pens at time of worldwide GLP-1 RA stock shortage. Further, this is a pilot trial designed to assess acceptability and tolerability of the intervention. As such, it will provide vital data for appropriate sample size calculation for future large scale efficacy trials.

Given the recognised barriers related to implementing lifestyle behavioural changes to reduce T2D risk in women with previous GDM and high cardiometabolic risk, additional approaches to managing long-term T2D risk are needed. Pharmacological interventions and physical activity monitors have the potential to reduce cardiometabolic risk within this patient population, but feasibility and tolerability of such interventions remain to be clarified. Trial outcomes will offer important insights and are crucial for evaluating the longer-term effectiveness and acceptability of the proposed interventions.

## Supplementary material

10.1136/bmjopen-2025-107551 online supplemental file 1

10.1136/bmjopen-2025-107551 online supplemental file 2

10.1136/bmjopen-2025-107551 online supplemental file 3

10.1136/bmjopen-2025-107551 online supplemental file 4

10.1136/bmjopen-2025-107551 online supplemental file 5
