# Supplementary material for: DIVINE–pilot trial: a phase 2 multicentre, randomised pilot trial of pharmacotherapy and physical activity monitoring conducted in women with recent gestational diabetes and increased risk of type 2 diabetes recruited from tertiary referral hospitals in Australia
Source: BMJ Open. 2025 Dec 12;15(12):e107551. doi: 10.1136/bmjopen-2025-107551 (PMC12706213; doi:10.1136/bmjopen-2025-107551)
Supplement: online supplemental file 2 [file bmjopen-15-12-s002.pdf]

Participant ID:

Study ID:

EQ-5D-5L

Health Questionnaire

English version for Australia

VERSION FOR INTERVIEWER ADMINISTRATION

Note to interviewer: although allowance should be made for the interviewer's particular style of speaking, the wording of the questionnaire instructions should be followed as closely as possible. In the case of the EQ-5D-5L descriptive system on page 2 of the questionnaire, the precise wording must be followed.

If the respondent has difficulty choosing a response, or asks for clarification, the interviewer should repeat the question word for word and ask the respondent to answer in a way that most closely resembles his or her thoughts about his or her health today.

INTRODUCTION

(Note to interviewer: please read the following to the respondent.)

We are trying to find out what you think about your health. I will explain what to do as I go along, but please interrupt me if you do not understand something or if things are not clear to you. There are no right or wrong answers. We are interested only in your personal view.

First, I am going to read out some questions. Each question has a choice of five answers. Please tell me which answer best describes your health TODAY.

Do not choose more than one answer in each group of questions.

(Note to interviewer: first read all five options for each question. Then ask the respondent to choose which one applies to him/herself. Repeat the question and options if necessary. Mark the appropriate box under each heading. You may need to remind the respondent regularly that the timeframe is TODAY.)

© 2021 EuroQol Research Foundation. EQ-5D™ is a trade mark of the EuroQol Research Foundation. Australia (English) v1.1

Participant ID:

Study ID:

EQ-5D DESCRIPTIVE SYSTEM

First, I would like to ask you about MOBILITY. Would you say that:

You have no problems with walking around?	❑
You have slight problems with walking around?	❑
You have moderate problems with walking around?	❑
You have severe problems with walking around?	❑
You are unable to walk around?	❑

Next, I would like to ask you about PERSONAL CARE. Would you say that:

You have no problems with washing or dressing yourself?	❑
You have slight problems with washing or dressing yourself?	❑
You have moderate problems with washing or dressing yourself?	❑
You have severe problems with washing or dressing yourself?	❑
You are unable to wash or dress yourself?	❑

Next, I would like to ask you about USUAL ACTIVITIES, for example work, study, housework, family or leisure activities. Would you say that:

You have no problems doing your usual activities?	❑
You have slight problems doing your usual activities?	❑
You have moderate problems doing your usual activities?	❑
You have severe problems doing your usual activities?	❑
You are unable to do your usual activities?	❑

Next, I would like to ask you about PAIN OR DISCOMFORT. Would you say that:

You have no pain or discomfort?	❑
You have slight pain or discomfort?	❑
You have moderate pain or discomfort?	❑
You have severe pain or discomfort?	❑
You have extreme pain or discomfort?	❑

Finally, I would like to ask you about ANXIETY OR DEPRESSION. Would you say that:

You are not anxious or depressed?	❑
You are slightly anxious or depressed?	❑
You are moderately anxious or depressed?	❑
You are severely anxious or depressed?	❑
You are extremely anxious or depressed?	❑

© 2021 EuroQol Research Foundation. EQ-5D™ is a trade mark of the EuroQol Research Foundation. Australia (English) v1.1

Participant ID:

Study ID:

EQ-5D VAS

- **Now, I would like to ask you to say how good or bad your health is TODAY.**

- **I would like you to picture in your mind a vertical line that is numbered from 0 to 100.**
  (Note to interviewer: if interviewing face-to-face, please show the respondent the VAS line.)

- **100 at the top of the line means the <u>best</u> health you can imagine.**

  0 at the bottom of the line means the <u>worst</u> health you can imagine.

- **I would now like you to tell me the point on this line where you would put your health TODAY.**
  (Note to interviewer: mark the line at the point indicating the respondent's health today. Now, please write the number you marked on the line in the box below.)

THE RESPONDENT'S HEALTH TODAY = ☐

Thank you for taking the time to answer these questions.

The best health
you can imagine

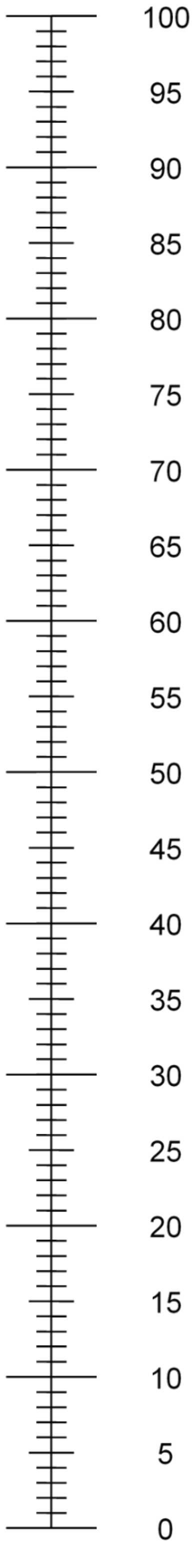

The worst health
you can imagine

© 2021 EuroQol Research Foundation. EQ-5D™ is a trade mark of the EuroQol Research Foundation. Australia (English) v1.1
