# Supplementary material for: DIVINE–pilot trial: a phase 2 multicentre, randomised pilot trial of pharmacotherapy and physical activity monitoring conducted in women with recent gestational diabetes and increased risk of type 2 diabetes recruited from tertiary referral hospitals in Australia
Source: BMJ Open. 2025 Dec 12;15(12):e107551. doi: 10.1136/bmjopen-2025-107551 (PMC12706213; doi:10.1136/bmjopen-2025-107551)
Supplement: online supplemental file 5 [file bmjopen-15-12-s005.pdf]

The George Institute for Global Health

Study ID: **Participant ID:**

**Acceptability of activity monitor questionnaire (End of intervention)**		
Date:		
1.	Did you find activity monitors helpful in improving your activity levels?	▪ Yes<br>▪ No<br>▪ Maybe
2.	Why or why not?	Free text
3.	Did you use any other devices or App to help you track your activity levels?	▪ Yes (go to Q4.)<br>▪ No (go to Q5.)
4.	What did you use?	Free text
5.	Do you have any other comments?	Free text

**Acceptability of activity monitor questionnaire (End of follow-up)**		
Date:		
1.	Did you use the activity monitor in the last 6 months?	▪ Yes (go to Q2.)<br>▪ No (go to Q3.)
2.	How often have you used the monitor?	▪ Every day<br>▪ 1-2 times a week<br>▪ 3-4 times a week<br>▪ 5-6 times a week<br>▪ Once every couple of weeks<br>▪ Once every month<br>▪ During exercise only<br>▪ Other (specify)
3.	Why not?	▪ Uncomfortable<br>▪ Don't find it helpful<br>▪ Didn't want to<br>▪ Other (specify)
4.	Did you find activity monitors helpful in improving your activity levels?	▪ Yes<br>▪ No<br>▪ Maybe
5.	Why or why not?	Free text
6.	Did you use any other devices or App to help you track your activity levels?	▪ Yes (go to Q7.)<br>▪ No (go to Q8.)
7.	What did you use?	Free text
8.	Do you have any other comments?	Free text
