# Supplementary material for: DIVINE–pilot trial: a phase 2 multicentre, randomised pilot trial of pharmacotherapy and physical activity monitoring conducted in women with recent gestational diabetes and increased risk of type 2 diabetes recruited from tertiary referral hospitals in Australia
Source: BMJ Open. 2025 Dec 12;15(12):e107551. doi: 10.1136/bmjopen-2025-107551 (PMC12706213; doi:10.1136/bmjopen-2025-107551)
Supplement: Supplementary file 6 [file bmjopen-2025-107551.reviewer_comments.pdf]

PEER REVIEW HISTORY

BMJ Open publishes all reviews undertaken for accepted manuscripts. Reviewers are asked to complete a checklist review form and are provided with free text boxes to elaborate on their assessment. These free text comments are reproduced below.

ARTICLE DETAILS

Title (Provisional)

DIVINE – Pilot Trial: A phase 2 multicentre, randomised pilot trial of pharmacotherapy and physical activity monitoring conducted in women with recent gestational diabetes and increased risk of type 2 diabetes recruited from tertiary referral hospitals in Australia

Authors

Chen, Angela Xun-Nan; Lee, Vivian Y.; Donges, Katherine; Giancas, Chris; Angell, Blake; Parmenter, Belinda; Barrett, Helen L; Henry, Amanda; Patel, Anushka; Arnott, Clare

---

VERSION 1 - REVIEW

Reviewer 1

Name Basri, Nurul Iftida

Affiliation Universiti Putra Malaysia Fakulti Perubatan dan Sains Kesihatan, Obstetrics & Gynaecology

Date 29-Sep-2025

COI None

This is an interesting study, comprising of multiple groups with and without intervention. However there is no group of control without any intervention to compare with. Would authors consider purely control group without metformin?

How will the participants be monitored for compliance, to metformin, semaglutide and physical activity throughout the study? Authors mentioned this during end of intervention visit, but will, this be checked at any other time?

When will they be considered as drop-out if non compliance happen?

How will the contraception of choice chosen by each participants?

How does the telehealth follow up takes place? will it be real time consultation? what about the measurement (BMI, blood pressure, heart rate), how will this be obtained during telehealth follow up?

VERSION 1 - AUTHOR RESPONSE

Reviewer:1
Dr. Nurul Iftida Basri, Universiti Putra Malaysia Fakulti Perubatan dan Sains Kesihatan

Comments to the Author:

This is an interesting study, comprising of multiple groups with and without intervention. However, there is no group of control without any intervention to compare with. Would authors consider purely control group without metformin?

We fully understand the premise of this question. We have not included a strict control arm (i.e. no intervention) as this is a pilot trial and the primary aim of the study is to assess the feasibility and tolerability of metformin and glucagon-like peptide 1 receptor agonists in this patient group. Based on the outcomes of this trial related to feasibility and tolerability – we agree, it will be a critical consideration in any larger, subsequent studies as to whether we include a control arm without any pharmacological intervention.

How will the participants be monitored for compliance, to metformin, semaglutide and physical activity throughout the study? Authors mentioned this during end of intervention visit, but will, this be checked at any other time?

We have clarified study follow-up and assessments to show that compliance to pharmacological and physical activity interventions will be reviewed at all telehealth visits (Weeks 4, 8, 12, 16, 20 and 24).

Lines 310 to 313 have been updated as follows:

- "This will enable assessment of pharmacological and physical activity intervention tolerability and compliance utilising the Brief Medication Questionnaire (Appendix 4) and Acceptability of activity monitor questionnaire (Appendix 5) respectively and provide opportunities to up-titrate semaglutide dose for applicable participants"

The telehealth visits that will be carried out throughout the trial to monitor compliance are also included in Table 1: Summary of study timeline and assessments.

When will they be considered as drop-out if non-compliance happens?

Participants will be considered drop-outs if they exit the study for whatever reason prior to the completion of the study (i.e. the end of study visit). As we are specifically assessing tolerability and acceptability of the study interventions, provided the participant continues to attend follow-up visits, non-compliance will not be viewed as a "drop-out" per se.

This has been clarified in Lines 381 – 382:

- "Dropout is defined as exit from the trial for whatever reason prior to the completion of the study (i.e. end of intervention visit)."

How will the contraception of choice be chosen by each participant?

Choice of contraception will be at each participant's choice, however, in order to be included in the study, all contraception used will need to be defined as "highly effective" contraception as outlined by the International Council for Harmonisation of Technical Requirements for Pharmaceuticals for Human Use.

This is outlined in Lines 193 – 198:

- "Individuals who are unwilling to use International Council for Harmonisation of Technical Requirements for Pharmaceuticals for Human Use defined "highly effective" contraception, unable to participate in physical activity or have known inability to give informed consent to participate due to severe active mental health issues or major developmental disability as identified through the maternity database will also be excluded from trial participation."

How does the telehealth follow up takes place? will it be real time consultation? what about the measurement (BMI, blood pressure, heart rate), how will this be obtained during telehealth follow up?

Telehealth consultations will take place via phone or video calls – these will take place in real time. Clinical physical measurements such as BMI, blood pressure and heart rate will not occur at these visits.

Rather these will be visits used evaluate intervention tolerability and provide additional advice on semaglutide up-titration for appropriate participants. Further, these visits will be used to ensure that urine pregnancy tests are conducted at regular intervals and are negative. Further clarifications have been made in Line 309 – 316:

- "Telehealth (telephone or video call) follow-up visits will be conducted at weeks 4, 8, 12, 16, 20 and 24. This will enable assessment of pharmacological and physical activity intervention tolerability and compliance utilising the Brief Medication Questionnaire (Appendix 4) and Acceptability of activity monitor questionnaire (Appendix 5) respectively and provide opportunities to up-titrate semaglutide dose for applicable participants. The occurrence of any study outcomes, and adverse events in all participants, as well as adherence to contraception and urinary pregnancy tests results in those randomised to metformin + semaglutide will be reviewed (Table 1)."
-
